# Opportunity makes a hub or a leader

**DOI:** 10.7554/eLife.105929

**Published:** 2025-02-12

**Authors:** Marjan Slak Rupnik

**Affiliations:** 1 Center of Physiology and Pharmacology, Medical University of Vienna Vienna Austria

**Keywords:** light-sheet microscope, pancreatic islets, calcium oscillation, β-cell, Mouse

## Abstract

Functional subpopulations of β-cells emerge to control pulsative insulin secretion in the pancreatic islets of mice through calcium oscillations.

**Related research article** Jin E, Briggs JK, Benninger RKP, Merrins MJ. 2024. Glucokinase activity controls peripherally-located subpopulations of β-cells that lead islet Ca2+ oscillations. *eLife* **13**:RP103068. doi: 10.7554/eLife.103068.

Pancreatic islets are specialized regions within the pancreas composed of endocrine cells responsible for producing, storing and releasing key metabolic hormones, including insulin. Insulin plays a crucial role in regulating cell metabolism. When stimulated, beta cells (also known as β-cells) within the pancreatic islets secrete insulin, which promotes anabolic metabolism and returns specific nutrients – including glucose – to the base level. This function is disrupted in conditions where blood sugar levels are either too high, such as diabetes mellitus, or too low.

Fluctuations in blood glucose levels change the flux of calcium ions in β-cells, which triggers insulin secretion. At stimulatory glucose levels, β-cells continuously switch between active and inactive phases, creating a pulsatile release of insulin that is crucial for maintaining metabolic balance. However, most studies of β-cell populations have been limited by an experimental design that could only image a two-dimensional single plane of the islets containing a small number of β-cells.

Now, in eLife, Erli Jin, Jennifer Briggs, Richard Benninger and Matthew Merrins of the University of Wisconsin-Madison and the University of Colorado Anschutz Medical Campus report using three-dimensional imaging techniques to study the activity of β-cell subpopulations in whole isolated mouse pancreatic islets (Jin et al., 2024). Tracking cell activity over time revealed that insulin secretion is regulated by a complex interplay of subpopulation networks and the fluctuating on-and-off activity of β-cells rather than by the specific traits of individual cells.

The analyses confirmed that highly synchronized cell hubs were located at the center of the islet, while the cells found at the periphery tended to switch on first. However, contrary to previous belief, different regions of the islet were able to initiate calcium waves, challenging the view that leader cells are the only subpopulation that drive the activity (Rutter et al., 2024).

Jin et al. provide strong experimental evidence that self-managing hierarchies emerge and drive the collective function of β-cell collectives while stripping individual β-cells from predetermined tasks of being leaders or hub cells (Korošak et al., 2021; Stožer et al., 2021). Consequently, a lack of correlations between specialized gene expression patterns of individual β-cells and their function would mean that a targeted disruption would not likely lead to organ dysfunction and diabetes (Rutter et al., 2024). Any previously observed correlations assigning fixed functions to individual β-cells over a prolonged period – even though statistically possible – are improbable and poorly reproducible in both high-resolution 2D studies and in the 3D study of Jin et al. (Postic et al., 2023).

Taken together, the findings suggest that the function of a β-cell in a collective is mostly determined by its location, environment and timing rather than its intrinsic molecular factors. The question is, therefore, not whether leaders or hubs exist. There will always be dynamic on/off regions and radially projecting central hubs during each calcium wave. And these hubs exhibit a migratory activity pattern that shifts between the waves, and changes at a timescale faster than protein expression levels can be altered in β-cells (Figure 1).

**Figure 1.**
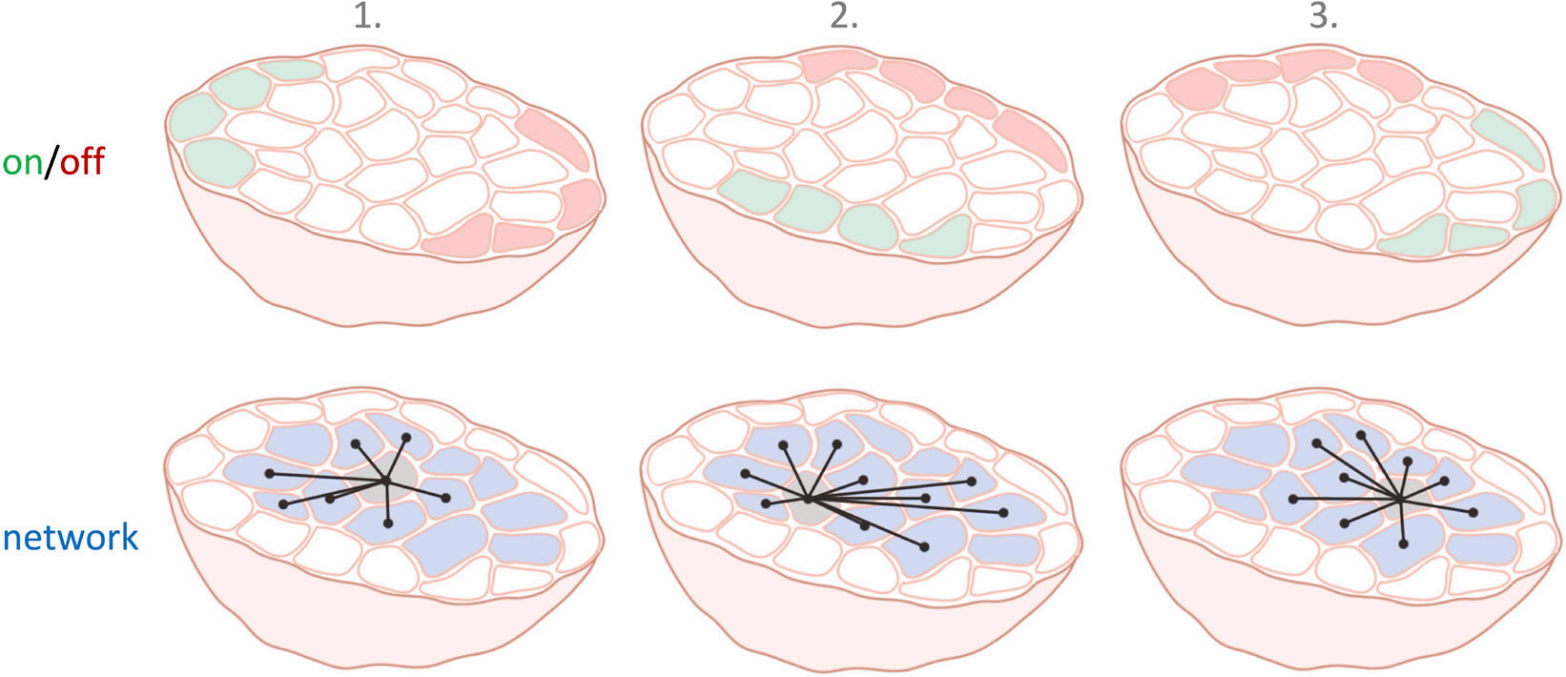
Cell subpopulations in the pancreatic islets in three consecutive calcium waves. The top row illustrates which β-cell subpopulations are switched on (green) and off (red) over three different calcium waves. The location of these on and off cells varies with each calcium wave. The bottom row shows the interconnected network structure of the centrally located current hub β-cell (light grey).

The lack of potential molecular determinism in β-cells aligns with the growing belief that across multiple levels of biological organization – from cells to organs – genomic information alone does not fully dictate cellular function, let alone cell-to-cell interactions (Ball, 2023). It is time to move beyond misinterpretations of Schrödinger’s determinism and embrace causal emergence with agency – for example, the ability of cell collectives to regulate themselves and their environment at every level (Schrödinger, 1967; Levin, 2021).

The pancreatic islet will remain a powerful experimental model for probing the intricate intercellular communications within an organ. Freed from the limitations of weak correlative statistics and reductionist specialist terminology, network science can reclaim its role in uncovering the emergent properties of β-cell collectives extending beyond insulin release (Gosak et al., 2018). A promising direction is the sensory function of the islets, where the number of β-cells and the physical dimensions of a typical islet would enable precise collective nutrient sensing that surpasses the sensory capacity of single β-cells. This would allow for a more accurate detection of a wide range of nutrients beyond plasma glucose (Fancher and Mugler, 2017).

Future studies will face the challenge of translating the architecture and function of mouse islets to their human equivalent. A different microarchitecture of the human pancreatic islet may reflect differences in β-cells activity and coordination, possibly reflecting a significantly lower metabolic rate in humans.
